# Integrated system for the proactive analisys of risk infection in patient’s surgical route

**DOI:** 10.1093/eurpub/ckac131.417

**Published:** 2022-10-25

**Authors:** E Montella, S Iodice, S Bellopede, A Frangiosa, A Giovagnoli, G Mazia, M Triassi

**Affiliations:** Public Healt Departement, Università degli Studi di Napoli, Naples, Italy

## Abstract

**Background:**

The most common hospital safety incidents are Hospital-Acquired-Infections (HAI) and among these Surgical Site Infections (SSIs). Our study proposes the use of a proactive system to manage risk combining the new Risk Identification Framework by WHO (IPCAF), the Lean method and the hospital’s Procedure Analysis. Each of the methods has pros and cons, and there is no existing literature that researches the concurrent use of all three methods. We focused on analysing patients’ surgical route to demonstrate that using an integrated system for preventing SSIs delivers enhanced results and significantly contibutes to a reduction in occurence of SSIs.

**Methods:**

We conducted a retrospective observational study from 18 March 2019 to 20 April 2019 at Azienda Ospedaliera Universitaria Federico II di Napoli, Italia (Europa). The study is structured in 3 phases:

Phase 1- application of proactive risk management tools (18 March- 15 April 2019);

Phase 2-integration of results with the elaboration of a single system for proactive risk management (15-20 April 2019);

Phase 3- collection of epidemiologic data concerning SSI. We used the incidence of surgical site as efficiency indicator (1-10 March 2022).

The endpoints identified were:

Primary Endpoint: a reduction of infection occurrence in surgical sites

Secondary Endpoint: identification of critical points and control points within the surgical process with relevant corrective measures

**Results:**

The rate of incidence of SSIs was selected as the efficacy indicator for the system. Our study recorded a 2.40% incidence rate for SSIs in 2020, compared to an incidence rate of 3.80% in 2018 and of 3.5% in 2017.

**Conclusions:**

Considering the economic impact of the infections, along with the increased incidence of mortality and morbidity, employing all available tools to try and reduce SSIs incidence becomes paramount. A small reduction can produce significant cost savings that can be invested in other prevention programs.

**Key messages:**

• Integrated system in proactively and promptly identifying risks related to patients’ surgical routes is effectiveness.

• The system can be adapted to different healthcare settings, to prevent adverse incidents by employing a risk management strategy, and to further enhance existing strategies.

